# Arousing the Sound: A Field Study on the Emotional Impact on Children of Arousing Sound Design and 3D Audio Spatialization in an Audio Story

**DOI:** 10.3389/fpsyg.2020.00737

**Published:** 2020-05-06

**Authors:** Francisco Cuadrado, Isabel Lopez-Cobo, Tania Mateos-Blanco, Ana Tajadura-Jiménez

**Affiliations:** ^1^Communication and Education, Universidad Loyola Andalucía, Seville, Spain; ^2^Department of Theory and History of Education, and Social Pedagogy, Universidad de Sevilla, Seville, Spain; ^3^DEI Interactive Systems Group, Department of Computer Science and Engineering, Universidad Carlos III de Madrid, Madrid, Spain

**Keywords:** audio-story, sound-design, 3D-sound, emotion, immersion, imagery

## Abstract

Sound from media increases the immersion of the audience in the story, adding credibility to the narration but also generating emotions in the spectator. A study on children aged 9–13 years (*N* = 253), using an audio story, investigated the emotional impact of arousal vs. neutral treatment of sound and 3D vs. stereo mix spatialization. The emotional impact was measured combining three different measures: physiological (Electrodermal activity), self-report (pre-post exposition), and richness of mental images elicited by the story (using Think-aloud technique after exposition). Results showed higher emotional impact of the arousal and 3D audio conditions with different patterns according to the age of the participants and distinctive types of interaction when both variables were combined.

## Introduction

For the majority of us, sounds are present in many aspects of our lives. They accompany many of our actions, such as opening a door or walking down the stairs; they signal the presence of other individuals, animals, objects, and even environmental events, such as a thunderstorm; and, through reflections in walls and other surfaces, sounds provide information of the geometry of the space we are in. Our auditory system, as the rest of our sensory systems, has evolved to monitor the surrounding environment, obtain information, and alert us of significant events so that we can adapt our behavior accordingly and keep safe (Graziano, 2001). In this respect, the auditory system is especially good at detecting changes and quickly orienting our behavior toward them; often, the auditory system acts faster than, for instance, vision does (McDonald et al., 2000). For this reason, the auditory system is often known as “a warning system” (Juslin and Västfjäll, 2008).

Hearing a sound will offer trigger an emotional response in listeners. Indeed, sounds can elicit a full range of emotional responses in listeners (Bradley and Lang, 1999, 2000; Juslin and Västfjäll, 2008). People can be startled by a sudden scream in the middle of the night, annoyed by the traffic noise, pleased by a bird song, or thrilled by hearing football crowds cheering. Emotional responses produce changes in our physiological state, behavior, and feelings, getting our body ready for action (e.g., Levenson, 1994; LeDoux, 1998; Seth, 2013).

Previous research investigating emotional responses to sound has mostly focused on trying to connect physical sound attributes, such as intensity, frequency, or the time structure of the sound signal (Schirmer et al., 2016), with basic emotional responses. For instance, equal pleasantness contours for tones varying in frequency and intensity have been developed (Todd, 2001), and a few studies have suggested a correspondence between sound intensity and emotional arousal since increasing loudness results in an increase in the orienting response (e.g., Sokolov, 1963; Lang et al., 1990). Also, correspondences between sound clarity (a parameter directly connected to the amount of high frequency in a sound) and the emotional valence has been found (Cho et al., 2001). Nevertheless, other studies have evidenced that looking at physical properties alone cannot fully capture emotional responses to sounds. For instance, the study by Landstrom et al. (1995) showed that only around 20% of noise-induced annoyance related to physical characteristics of the noise (see also Bjork, 1999; Bradley and Lang, 2000). In another study, everyday sounds were used (e.g., a cow and a rollercoaster), but the identification of these sounds was impaired by using a neutralization algorithm that preserved the physical properties of the sounds; this was done in order to show that only 20–25% of the emotional responses to these everyday sounds depended of the physical properties of the sound (Asutay et al., 2012).

The studies above have suggested that listeners do not react emotionally just to acoustic waves but also to sound sources and sound events, and that emotional responses to sound depend on the interpretation and meaning (i.e., relevance) the listeners attribute to these particular sound sources and events (Jäncke et al., 1996; Gygi, 2001; Juslin and Västfjäll, 2008; Tajadura-Jiménez, 2008). Furthermore, this interpretation is the result of an interaction between the sound itself, the context of when and where sound is heard, and the listener (Blauert and Jekosch, 1997; Jekosch, 1999). Therefore, when studying emotional responses to sound, it is important to consider other variables apart from the physical properties of the sound. These variables relate to whether sounds can be identified as objects or events (Jäncke et al., 1996; Bradley and Lang, 2000; Asutay et al., 2012); the context, such as the events that preceded the sounds, the presence of other multisensory events, or the space where the sound is heard (Västfjäll et al., 2002; Tajadura-Jiménez et al., 2010; Berger et al., 2018); and the individual differences of listeners. The same sound may be interpreted in a substantially diverse manner by different listeners; listeners may vary in their previous experiences, expectations, personality traits, or individual goals (Grimshaw, 2014); therefore, the same sound may elicit a rather different emotional response in different listeners (social and cultural memory; Tajadura-Jiménez, 2008).

All these changes affect attention, cognitive, and perceptual processes (De Gelder and Vroomen, 2000) and influence our judgments and decisions (Peters et al., 2006). As a result, it is common 4 that sound is used in products or media applications in order to transmit information, grab the attention of users, or influence their attention. Sound and music from different types of media products (film, TV series, documentaries, podcasts, and videogames) contribute to the success of the audience experience, adding credibility to the created story, making the narration more understandable, and also generating emotions in the spectator. Thanks to the veridiction pact (Zunzunegui, 1995) and the semi-conscious perception of sound (Murch, 2001), sound design has the power to increase the immersion and participation of the audience in the story.

Regarding audio-visual media products, different studies have analyzed the emotions elicited by sound as part of a media narration, considering the presence or absence of sound and musical narrative elements. These studies have found a greater response in EDR (electrodermal response), heart rate, and temperature in stimuli with sound effects compared to silence (Shilling et al., 2002); also exhibited were a significant increase in EDA and questionnaires in stimuli with sound compared to silence (Scorgie and Sanders, 2002). Also, a better performance in the accomplishment of a task (driving game) has been achieved when the music is selected by the participant. Taking into consideration the diegetic vs. non-diegetic approach, Grimshaw (2008) found that diegetic sound provides a higher level of immersion, while music increases immersion and reduces tension and negative affect.

Focusing on the audio-only kind of media products, such as radio programs, podcasts, or audio narrations, one of the few studies with children in this field concluded that the use of narration, character's direct voice, and sound effects in an audio story generated more enjoyment, attention, and positive emotional impact in children aged 3–4 years (Ritterfeld et al., 2005). A slightly different approach in the emotional impact of media sound is the consideration of the relationship between the narration (voice over or dialogues), sound effects, and the use of different sound shots (the placement of sound in several distances from the listener perspective). In a study focused on the analysis of mental images and attention level in sound fictional stories, Rodero (2012) compared four versions of the same stimulus: (1) narration, (2) narration with sound effects, (3) narration with different sound shots, and (4) narration with sound effects and different sound shots. Results showed a higher level of creation of mental images and attention in stimulus with sound effects vs. stimulus without sound effects. Furthermore, the use of different sound shots in the narration also derived a higher level of creation of mental images and attention compared to narration without the use of sound shots. Finally, the highest level of creation of mental images and attention was found in stimulus that included narration, sound effects, and the use of different sound shots.

As these last findings suggest, space and spatial localization of sound is one of the key elements that increase the immersion and emotional impact on the listener (Murphy and Pitt, 2001). These findings are consistent with Steele and Chon (2007), who found that the spatial location of a virtual sound object, although currently limited in terms of game implementation, has a significant potential related to the emotions.

A further key element related to the listening experience and, more specifically speaking, to the spatial dimension of sound is that the choice of headphones or speakers could be a significant contextual variable (Cox, 2008; Hong et al., 2017), particularly in terms of location and immersion (Grimshaw, 2007) and emotional impact. In a comparable study, Murphy and Pitt (2001) showed a preference for the use of headphones, arguing that it “…allows the designer to incorporate more complex sound objects whose subtleties will not be lost due to background noise, speaker conversation, etc.” Headphones seem to produce a more immersive experience, and the commercial availability of a wide range of headphones (many designed specifically for computer games) suggests that the use of headphones is common in a player's natural environment (LaGrou, 2014). These studies provide evidence of how the spatial dimension of sound, in this case related to the use of headphones, may impact on immersion and emotional impact.

## Objective and Hypotheses

The aim of the present study was to investigate the potential impact of “emotionally marked” sound effects and of 3D spatialization on emotional responses and quality of mental images elicited in children when listening to an audio story. According to Valkenburg and Beentjes (1997), a story presented in auditory form is expected to stimulate imagination and fantasy in children.

This study is part of the research project “Unconscious listening,” which is focused on the analysis of the emotional impact of sound in children and its possibilities to increase and improve learning in the scholar environment. As stated by Ritterfeld et al. (2005), audio stories might support cognitive and emotional development in children. Following the research design of the “Unconscious listening” project, the study focused on Primary and Secondary Education children. Although no references have been found in previous studies about differences in the emotional impact of sound in children from distinctive ages, this has been considered in the present study, according to the various educational level of participants.

According to the previous findings, several hypotheses were formulated:

**H1**: the use of “emotionally marked” (i.e., arousing vs. neutral) sound in the design and production of a sound story will elicit more intense emotional responses in the listener compared to a sound story without this emotional manipulation in its design and production.

**H2**: the use of “emotionally marked” sound in the design and production of a sound story will generate richer and more detailed mental images in the listener than a soundtrack without this emotional intention.

**H3**: a soundtrack mixed in 3D sound format will elicit a more intense emotional response in the listener compared to a soundtrack mixed in stereo.

**H4**: a soundtrack mixed in 3D sound format will generate a greater number of mental images as a well as richer and more detailed mental images in the listener compared to a soundtrack mixed in stereo.

**H5**: the emotional impact elicited by both emotional marked sounds and/or 3D sound mix format takes place at an unconscious level and therefore will not be reported by the listener.

**H6**: the emotional impact and the mental images generated by both emotional marked sounds and/or 3D sound mix format exhibit different effects according to the educational level of the participants.

## Method

### Sample Description

The participant sample consisted of 253 children from two schools in Seville: Ntra. Señora del Águila (SSAA) and San José SSCC (PPBB). The participants were students from two educational levels: 128 participants from 4th Primary Education (9–10 years old) and 125 participants from 1st of Secondary Education (12–13 years old). Once the participation of each school in the project was agreed with schools' administrators, the sample selection was made by voluntary participation of students from the different school classes.

#### Ethical Implications

Participation in the project did not involve any physical or psychological risk to participants. Participants and their parents were conveniently informed about the whole project, and they signed informed consent forms before taking part in the study. All information collected followed the necessary protocols to safeguard the privacy and confidentiality of participants. The collected data were only used for the purposes of this research; the data were also protected so that only researchers could access it. The experiment was conducted in accordance with the ethical standards laid down in the 1964 Declaration of Helsinki, as revised in 2008, and approved by the Ethics Committee of the Universidad Loyola Andalucía.

### Stimuli

The stimulus consisted of a sound-only story (similar to a fictional radio story), based on an existing written story suitable for children between 9 and 14 years old: “*Los cohetes tienen forma de flauta*” (“*The rockets are flute-shaped*”). Due to the length of the whole tale, the two first chapters were selected to create the stimulus, resulting in a story length of 1860 words. The original text was adapted to produce a radio story. The adaptation basically consisted of increasing the number of character interventions and dialogues and reducing the amount of voiceover narration. According to Rodero (2012) a dramatized story generates a greater level of imagery and involvement in the listener compared to a narrated story. The narration and dialogues voices were recorded in a studio: a professional voice-over actor performed the role of the narrator, while two children with acting experience (a boy and a girl, aged 11 and 9, respectively) performed the role of the two characters of the story: Salva, a 10-year-old boy, and his 8-year-old sister, Elena.

According to the previous findings in research literature and the study hypothesis, the design of the stimuli centered around two independent variables: arousal level of sound design and sound spatialization. The arousal level of sound design was developed mainly through sound effects and ambiences. Two sound design proposals were elaborated: neutral and arousal marked. The sound treatment applied to every condition was based on very specific and subtle modifications of sound instead of on the presence or absence of certain types of sound (presence or absence of sound effects, dialogue, or music), which has been the approach of previous studies. The neutral condition consisted of sound effects and ambiences that, according to the description of the story, the locations, and the characters' action, movements, and dialogues, could be heard in a real-world situation. Also, a global equalization was applied to all the neutral sound effects and ambiences tracks, rolling off frequencies below 13 Hz and over 5.6 KHz (in both cases with 12 dB/octave slopes), in order to subtly reduce the clarity of the sound (related to a higher emotional elicitation, Cho et al., 2001) and the low-frequency impact.

The arousal-marked condition combined two distinctive approaches—sound parameter modification and sound source modification—which resulted in different procedures. Following the first approach, in certain cases, the same sound effects or ambiences from the neutral version were used but with changes in certain sound parameters. The modifications included equalization, modifying the high or the low frequencies of the sound in each case; changes in pitch to increase the clarity of the sound or to create a sensation of movement within the sound; changes in the loudness of specific sound effects; and added reverberation to increase the spaciousness of a specific sound. Following the second approach, some of the neutral sound effects and ambiences were substituted by others, looking for sound elements that supported a movement or an action, which illustrated a description or enriched a location. Special attention was given to the fact that both versions should include the same amount of sound elements, in the same moments of the story in order to provide to all the listeners a comparable sound story (avoiding the risk of obtaining different results based solely on the presence or absence of elements).

Furthermore, the strength of the variances in sound treatment between conditions was focused on moments of the story where it could be more narratively and dramatically effective, according to the development of the action throughout the story. Regarding arousal treatment, there were specific moments of the story that were identified to allow for a clearer difference between the sound design in the neutral vs. arousal conditions. Sound excerpts of these moments have been attached to this article, in both versions (neutral and arousal marked), as examples of the treatment applied in each case. These moments have been included here.

- “Meteor fall” (Supplementary Audios 1, 2: meteor_fall_neutral.mp3 and meteor_fall_arousal.mp3): the neutral version of the stimulus included a “woosh” sound to illustrate this fall. The arousal version included a denser “woosh” sound, with more high and low frequency components, as well as added reverberation.
- “Tic-tac”: While Salva says “*the time is relative*,” the neutral condition used the sound of the footsteps of the boy in the room. The arousal version used the sound of a reverberant tic-tac sound that, at the end of the sentence, becomes progressively slower until it stops.
- “Chronometer” (Supplementary Audios 3, 4: chronometer_neutral.mp3 and chronometer_arousal.mp3): while Elena plays the music on the flute, the sound of a chronometer counts the time she takes to play it. The neutral condition included the normal sound of a chronometer with no modifications. The arousal version modified this same sound, increasing the pitch and speed of the sound to make it higher pitched and to synchronize the tempo of the tic-tac sound with the tempo of the music Elena plays).
- “Down” effect: This effect is introduced to finish the moment in which Salva imagines the speed of his sister playing the flute while she is on a skateboard, and the narrator says “*the experiment was a total failure.”* In the neutral condition, the usual sound of children movement was used. In the arousal condition, the whole sound ambience that is listened while Salve speaks is pitched down until all the sounds disappear in a very low frequency register.

The sound treatment of most of these moments is consistent with previous studies, focusing in the modification of the parameters volume (Sokolov, 1963; Lang et al., 1990) and frequency spectrum (Cho et al., 2001), where it has been reported a higher emotional impact of sounds with great amount of high-frequency content.

**Figure 1:**
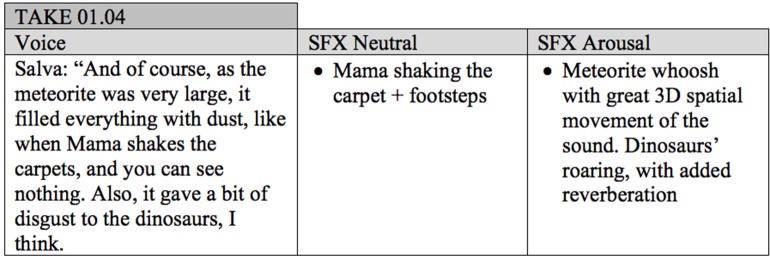
Excerpt of the sound design script.

Figure 1 shows an excerpt of the sound design script used during the stimuli production, specifically from one of the selected moments for arousal intervention: “meteor fall.”

**Figure 2:**
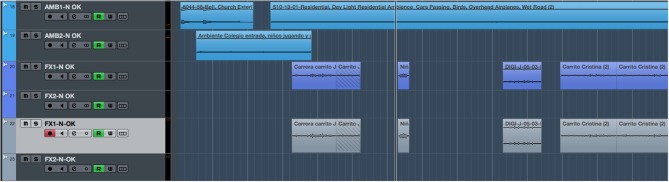
Screen capture showing the sound effects and ambiences used in the Neutral version for the first minute of the story.

**Figure 3:**
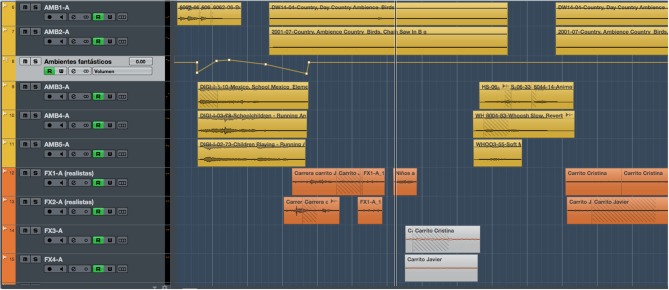
Screen capture showing the sound effects and ambiences used in the Arousal version for the first minute of the story.

Figures 2, 3 refer to the same timeline period (the first minute of the story). These figures display two screen captures of the tracks, sound effects, and ambiences used for each condition: neutral (Figure 2) and arousal (Figure 3). The number of audio tracks and the amount of sound layers are greater in the arousal version. For instance, a combination of different sound effects was used to recreate the gabble of children when leaving school. Also, two distinctive ambiences were layered to design a richer sound that recreated the environment of the village where the characters live.

**Figure 4:**
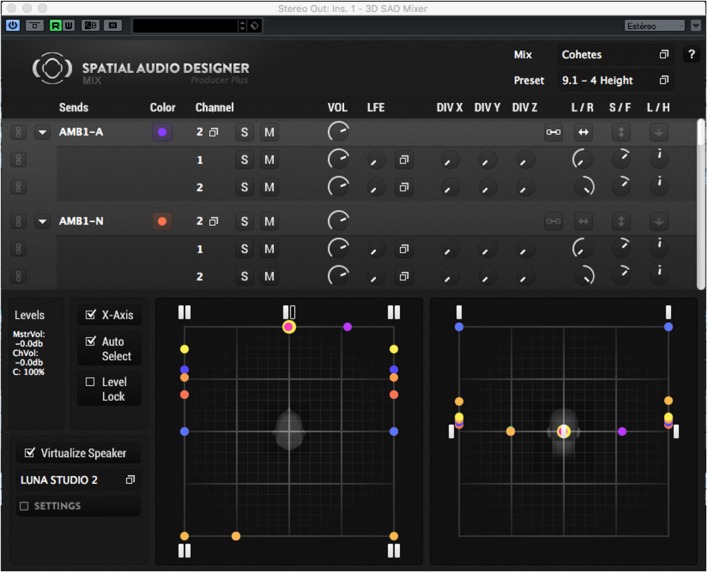
Screen capture of the 3D sound mix state in a specific moment of the story when sound elements are positioned and moved across the 3D field (horizontal and vertical).

Regarding sound spatialization, two versions of the stimulus were prepared, one mixed in stereo format and the other one mixed in surround 3D format. Both versions were produced to be listened through headphones. Nuendo 7.1 (from Steinberg) and Spatial Audio Designer (from New Audio Technology) were used to produce both mixes. The same recorded dialogue tracks were used in both mixes, keeping the volume and the clarity between the stereo and the 3D audio versions. The creation of the soundtrack followed the usual sound design and postproduction processes involved in film and media sound production: Foley effects recording and sound effects libraries were the main sound sources. All the ambiences and sound effects were edited using Nuendo 7.1. Different equalization, dynamic, and modulation plugins were used to process the sound, including several types of reverberation to recreate accurately the singular spaces represented in the story. From all the edited audio material (dialogues, sound effects, and ambiences), two separate mixes were produced. The stereo mix used the left-right panning, as well as volume, equalization, and reverberation to simulate the different spaces, position, and movement of sound sources. The 3D sound mix was produced with a 9.1 surround configuration (compatible with Dolby Auro 3D systems): five main channels, four elevated channels, and one LFE channel. Apart from the use of volume, equalization and reverberation, the spatial recreation was achieved through the movement of the sound objects in the 3D audio space (combining the front–back, left–right, and up–down axis). All the mixes (stereo and 3D) and parameter automation were done using the Spatial Audio Designer plugin, keeping the same peak and RMS levels between the two mixes, controlling in detail the clarity and understandability of dialogue in both versions. The final mixes in both formats were produced using the Headphone Surround 3D technology from the Spatial Audio Designer software. This technology consists of a binaural simulation of various mixing studios with multiple virtual loudspeaker arrays; this makes it possible to produce 3D mixes using all three dimensions and different locations and to listen to these 3D mixes using a pair of stereo headphones. Figure 4 shows a screen capture of the 3D sound mix configuration and sound object position in one of the specific moments of the story when sound elements are positioned and moved across the 3D field (horizontal and vertical). Each dot corresponds to a separate sound object (specific sound effects and ambiences). The master audio files produced with this software are compatible with any .wav or .mp3 file format player, and the 3D spatialization can be listened in any device using a pair of normal stereo headphones.

Although, in the 3D mix conditions, the whole sound was mixed using this spatial conception of space, there were also specific moments in the story that allowed for a clearer movement of sound through the three dimensional space. Sound excerpts of these moments have been attached to this article, in both versions (stereo and 3D), as examples of the treatment applied in each case. It is recommended to listen to the examples with headphones in order to notice the differences in the spatialization:

- “Salva dressing as a scientist” (Supplementary Audios 5, 6: scientist_st.mp3 and scientist_3D.mp3): Salva moves around the room to look for elements to dress himself like Albert Einstein. The 3D treatment combines the use of footsteps and the dialogue of Salva moving around the 3D space (following a specific path from center to left, to the back side of the room, to the right back of the room, and coming back to the front) together with specific sound effects (moving objects, manipulating boxes and household items, etc.) located in several specific places in the 3D space where Salva “stopped” to look for more elements to be use in his dressing.
- “Sad day for light-speed”: Salva speaks about how his sister is going to surpass light-speed with her flute performance over the skateboard. The 3D condition included a reverberant three-dimensional sound ambience that included the sound of a tower bell, thunder, the clapping and shouts of a large audience, and a high pitch “woosh” moving from the back left to the front right of the virtual sound space.
- “Skate-fall” (Supplementary Audios 7, 8: skate-fall_st.mp3 and skatefall_3D.mp3): Elena throws on the ramp with the skateboard while trying to play the flute. Different sound elements were located and moved through the 3D space: the sound of Elena screaming from left back to right front, the shouts of Salva from the very back left bottom; an intense wind sound and the rolling of the skateboard, also moving in the same direction of Elena's voice; and the final crash at the front right.

The sound treatment of most of these moments is also consistent with previous studies that consider spatial localization of sounds to be one of the key elements that increase the immersion and emotional impact on the listener (Murphy and Pitt, 2001; Steele and Chon, 2007). Furthermore, the use of binaural simulation of surround sound through headphones coincides with the conclusions of different authors (Murphy and Pitt, 2001; Grimshaw, 2007; Cox, 2008; Hong et al., 2017) about the increase of the location of sound sources and its relation to the immersion and emotional impact.

The combination of both variables (neutral vs. arousal and stereo vs. 3D) resulted in four distinctive sound conditions:

- Sound condition A: neutral sound design + stereo mix
- Sound condition B: neutral sound design + 3D sound mix
- Sound condition C: arousal sound design + stereo mix
- Sound condition D: arousal sound design + 3D sound mix

All the final stimuli were produced in wav file format with a 44.1 Khz sample rate and 16-bit depth to retain the standard of most consumer audio file formats.

### Measures

The emotional impact was measured by combining three different approaches: the physiological response (electrodermal response during exposition to the stimulus), self-reported emotional state (Self-Assessment Mannequin test—SAM, pre- and post-stimulus, and an immersion questionnaire, post stimulus) and mental images elicited by the story (specific questions and verbal expression of participants, using the Think-aloud technique after exposition). All the technical details of the equipment used are presented as an appendix at the end of this article (Appendix 1: Apparatus).

Below are the three different approaches:

- Physiological response: the electrodermal response (EDR) linked to emotional arousal or emotional intensity (Boucsein, 2012; Venables and Christie, 1973) was measured, providing the EDR per second for each participant.
- Self-reported emotional state: the Self-Assessment Mannequin test [SAM (Bradley and Lang, 1994)] was used to register the self-perception of each participant's emotional state. The SAM test is a picture-based instrument that measures three dimensions of a perceived emotion: valence (positive–negative), arousal (passive–active), and dominance (dominated–dominant). Only the two first dimensions (valence and arousal) were used in the study. The test was administrated to each participant before and after the exposition to the stimulus. The instrument was specially designed for this study to make possible that participants fulfilled the test using the same touchscreen of the tablet in which all the other activities were programmed (see the “Procedure” sub-section).
- To measure the mental images elicited by the story and the verbal expression of these emotions, a series of questions were designed to guide the participant through the exploration and verbalization of these mental images. Some questions were designed to be answered choosing an option (closed questions), while other questions were designed to register the verbal expressions of participants, using the Think-aloud technique (voice recording of participant's speech, in this case, as answers to specific questions).

**Figure 5:**
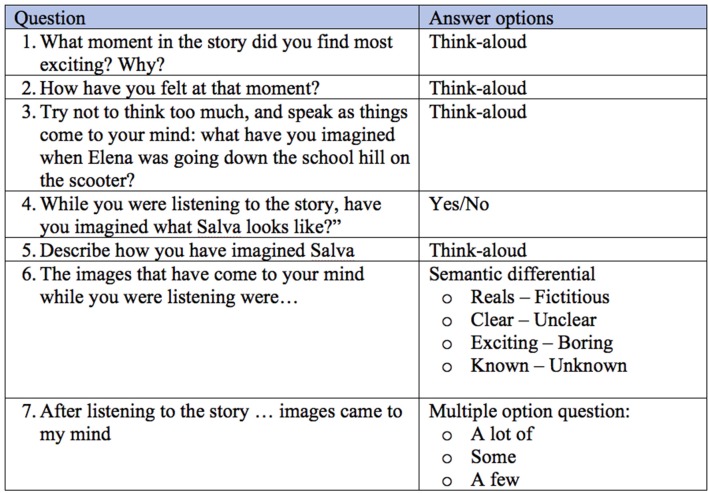
Questions and type of answer options for the mental images register.

The questions proposed to participants are shown in Figure 5.

First, we tested whether the distributions of the obtained data were normal using the Shapiro-Wilk test. None of the variables passed the normality test. Nevertheless, Q-Q plots showed moderate deviations from normality. Given that parametric statistical tests (ANOVAs) are quite robust to moderate deviations from normality (e.g., McDonald, 2014), in our analyses we opted for the use of both non-parametric Kruskal-Wallis tests for the four sound conditions, and the Analyses of Variance (ANOVA) tests allowed for the testing of the interactions between the factors sound emotion condition (Neutral, Emotional) and sound spatiality condition (stereo, 3D). The analyses were carried out using the SPSS software, version 24. Significant effects were followed by Mann-Whitney analyses (non-parametric tests of independent samples).

A qualitative analysis of the data obtained through Think aloud was carried out by three independent analysts. A total of 1,152 recordings were collected from the students who answered the questions they were asked. An analysis of the content of all the collected information was made, and an emergent categorical system was drawn up by the research team in which several dimensions of analysis were identified: (1) representation of the story, (2) elicited emotions, (3) mental images, and (4) immersion.

To develop the descriptive phase of the analysis, a total of eight categories and 25 subcategories were defined, identified from the described events and emotional states expressed. For the coding and subsequent qualitative analysis of the collected data, Nvivo 11PRO software was used, providing the open coding (Flick, 2012) of the information. Subsequently, an analysis of the frequency of references made in the responses to each of these categories and subcategories was carried out, and, based on these data, the interpretative phase of the analysis was carried out.

The variables contemplated in the analysis of the different measures were the sound condition (A, B, C, or D) and educational level (to consider age differences).

### Procedure

The field study was conducted over 2 weeks during school hours. A special classroom was prepared to give all the participants with enough space so as not to disturb each other while listening to the story, answering the questionnaires, or recording their voices during the Think-aloud tasks. The four versions of the stimulus (corresponding to the four sound conditions) were randomly assigned to each participant, and so none of them were aware of the listened version of the story. The version, gender, educational level (age), and school distribution of the stimuli is detailed in Table 1.

**Table 1:** Sample description and stimuli assignation.

**Stimuli version**	**Condition A** **(Neutral-St)**	**Condition B** **(Neutral-3D)**	**Condition C** **(Arousal-St)**	**Condition D** **(Arousal-3D)**
N° Participants	60	62	63	68
N° Male	33	27	35	36
N° Female	27	35	28	32
Educational level: 4° EP	29	32	32	35
Educational level: 1° ESO	31	30	31	33
N° participants SSAA	19	21	22	22
N° participants PPBB	41	41	41	46
N° Male SSAA 4° EP	5	7	6	4
N° Male SSAA 1° ESO	5	4	5	6
N° Male PPBB 4° EP	12	8	16	12
N° Male PPBB 1° ESO	11	8	8	14
N° Female SSAA 4° EP	3	4	5	6
N° Female SSAA 1° ESO	6	6	6	6
N° Female PPBB 4° EP	9	13	5	13
N° Female PPBB 1° ESO	9	12	12	7
4° EP SSAA	8	11	11	10
4° EP PPBB	21	21	21	25
1° ESO SSAA	11	10	11	12
1° ESO PPBB	20	20	20	21

## Results

### Effects on Physiological Arousal (EDR)

The measurement tool analyzed phasic activity related to emotion, i.e., the electrodermal response (EDR). The measurement unit was the electrodermal resistance in Kiloohms (KΩ) of each participant. All participants were exposed to a conditioning stimulus before the exposition to the studio stimulus with the purpose of accommodating them to the listening conditions and also to establish an individual baseline in the EDR response for each participant. Al collected data were preprocessed, subtracting the individual baseline level to all the measures for each participant. For the analysis of EDR, an initial ANOVA on the mean EDR values during the stimulus duration with sound condition (A—Neutral stereo, B—Neutral 3D, C—Emotional stereo, and D—Emotional 3D) as between-subjects variable was conducted. This analysis did not yield any significant results (*p* > 0.05). Furthermore, there were no significant differences in EDR between groups, as confirmed by a Mann-Whitney test (*p* > 0.05).

A subsequent analysis was conducted that looking only at the moments with special sound manipulations according to what has been exposed in the stimulus subsection:

- Meteor fall
- Dressing as a scientist
- Tic-tac
- Chronometer
- Sad day for light-speed
- “Down” effect
- Skate-fall

For each of the stimuli in Table 2, the maximum EDR value during the stimulus duration was calculated (Martin and Venables, 1980; Boucsein, 2012). Stimuli were all longer than or exactly 5 s long (note that, according to Edelberg, 1967, the EDR may be extended up to 5 s after the onset of the stimuli).

**Table 2:** Peak EDR ± SE values [kΩ] for each educational level (4th EP and 1st ESO) and sound condition (Neutral stereo, Neutral 3D, Emotional stereo, and Emotional 3D) for the different events (stimuli).

	**Stimuli version**	**Condition A**	**Condition B**	**Condition C**	**Condition D**
4° EP	Meteor fall	0.912 (0.92)	1.027 (0.88)	4.115 (0.88)	0.78 (0.85)
	Dressing as scientist	3.176 (1.03)	3.394 (0.98)	1.794 (0.98)	4.19 (0.95)
	Tic-Tac	0.826 (0.90)	2.23 (0.86)	3.4 (0.86)	0.793 (0.83)
	Chronometer	1.778 (0.97)	1.628 (0.92)	3.543 (0.92)	1.726 (0.89)
	Sad day for light-speed	1.939 (0.99)	3.008 (0.94)	3.356 (0.94)	3.195 (0.91)
	Down effect	0.84 (0.83)	0.607 (0.79)	2.796 (0.79)	0.731 (0.77)
	Skate-down	1.707 (1.1)	1.859 (1.04)	2.293 (1.04)	2.846 (1.01)
	**MEAN 4°** **EP (SD)**	1.638 (0.79)	1.836 (0.99)	2.762 (1.08)	2.001 (1.29)
1° ESO	Meteor fall	1.332 (0.90)	1.54 (0.91)	0.859 (0.89)	0.956 (0.87)
	Dressing as scientist	2.952 (0.99)	3.531 (1.01)	3.046 (0.99)	2.498 (0.96)
	Tic Tac	1.317 (0.87)	2.389 (0.89)	0.878 (0.87)	1.887 (0.84)
	Chronometer	2.602 (0.93)	1.816 (0.95)	2.578 (0.93)	2.348 (0.91)
	Sad day for light-speed	2.107 (0.96)	3.317 (0.97)	3.158 (0.96)	3.196 (0.93)
	Down effect	0.843 (0.80)	1.186 (0.82)	1.752 (0.80)	0.896 (0.78)
	Skate-down	2.231 (1.06)	4.196 (1.08)	2.639 (1.06)	5.121 (1.03)
	**MEAN 1°ESO (SD)**	1.801 (0.78)	2.378 (1.18)	2.025 (0.95)	2.224 (1.45)
	**MEAN GLOBAL (SD)**	**1.72 (0.76)**	**2.11 (1.09)**	**2.39 (1.06)**	**2.11 (1.33)**

*Mean values (SD) for 4th EP and 1st ESO are marked in bold font*.

The peak EDR values for each course and sound condition (A—Neutral stereo, B—Neutral 3D, C—Emotional stereo, and D—Emotional 3D) are displayed in Table 2. Peak EDR values were used as dependent variables for a Multivariate Analyses of Variance (MANOVAs) with between-subject factors course (4th EP and 1st ESO), sound emotion condition (Neutral and Emotional), and sound spatiality condition (stereo and 3D). Wilks' Lambda was used as the multivariate criterion. The results of the multivariate test revealed that there was a non-significant tendency, indicating an interaction between sound emotion condition and sound spatiality condition [*F*_(7, 238)_ = 1.90, *p* = 0.07, Wilks' Lambda = 0.947]. This interaction is explained by the results showing that, while for the neutral conditions there was an increase in EDR peak value from stereo to 3D (conditions A and B), for the emotional conditions, the difference between stereo and 3D conditions was smaller with a slight decrease in EDR from condition C to D (see mean values for conditions in Table 2).

**Figure 6:**
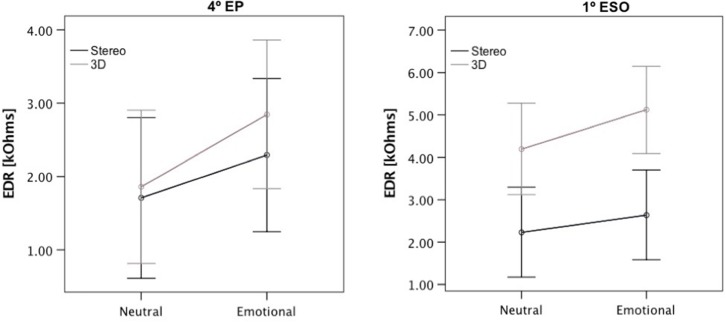
Peak EDR ± SE values [kΩ] for stimuli Skate-Down for each educational level (4th EP and 1st ESO) and sound condition (Neutral stereo, Neutral 3D, Emotional stereo, and Emotional 3D).

Univariate tests for each of the stimuli did not reveal a significant effect for any of the stimuli, but we observed tendencies toward an effect of the sound spatiality condition for the stimulus “skate down” [*F*_(1, 244)_ = 2.98, *p* = 0.085] with an overall larger peak EDR for the 3D version vs. the stereo version; for this stimulus, there was also a tendency in the effect [*F*_(1, 244)_ = 3.38, *p* = 0.067], and 1st ESO students displayed an overall larger peak EDR for this stimulus (see Figure 6). There was also a non-significant tendency toward an interaction between sound emotion condition and sound spatiality condition for the stimulus “tic tac” [*F*_(1, 244)_ = 2.77, *p* = 0.097], see Table 2.

### Effects on Self-Reported Emotional State (SAM)

**Figure 7:**
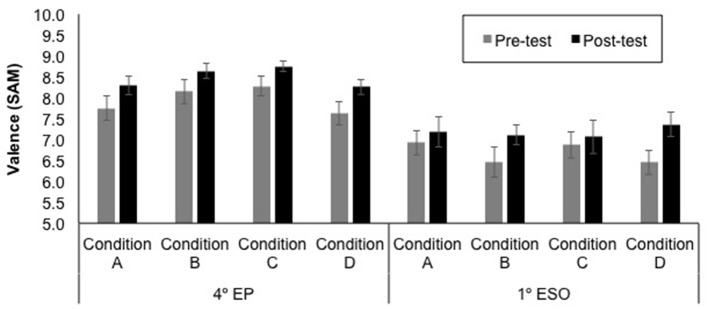
Mean valence ratings ± SE (on a nine-point scale) for each educational level (4th EP and 1st ESO), test time (pre- and post-experience) and sound condition (A—Neutral stereo, B—Neutral 3D, C—Emotional stereo, and D—Emotional 3D).

**Figure 8:**
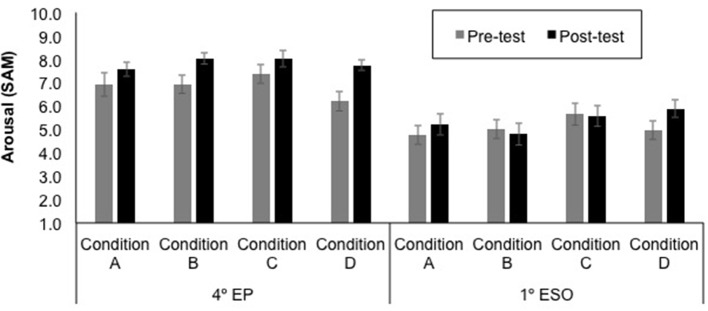
Mean arousal ratings ± SE (on a nine-point scale) for each educational level (4th EP and 1st ESO), test time (pre- and post-experience) and sound condition (A—Neutral stereo, B—Neutral 3D, C—Emotional stereo, and D—Emotional 3D).

The mean self-reported valence and arousal ratings for each educational level, test time (pre- and post-experience), and sound condition (A—Neutral stereo, B—Neutral 3D, C—Emotional stereo, and D—Emotional 3D) are displayed in Figures 7, 8. Note that higher ratings of valence and arousal represent more pleasant and arousing experiences, respectively.

First, both for self-reported valence and arousal ratings, the difference from pre-test to post-test were entered into Kruskal-Wallis tests, one for each educational level, to investigate potential variations in the pre-post change between the four sound conditions (A—Neutral stereo, B—Neutral 3D, C—Emotional stereo, and D—Emotional 3D). These analyses showed no significant differences between conditions (all ps > 0.05).

Then, in order to test the potential influence of the factor test time, sound emotion condition, and sound spatiality condition in the self-reported emotional state, according to the educational level, self-reported valence and arousal ratings were used as dependent variables for a Multivariate Analysis of Variance (MANOVA). The within-subject factor was test time (Pre vs. Post) and the between-subject factors were educational level (4th EP, 1st ESO), sound emotion condition (Neutral, Emotional), and sound spatiality condition (stereo, 3D). Wilks' Lambda was used as the multivariate criterion. The results of the multivariate test revealed that there was a significant main effect of educational level [*F*_(2, 231)_ = 46.36, *p* < 0.001,Wilks' Lambda = 0.71], a significant main effect of test time [*F*_(2, 231)_ = 14.39, *p* < 0.001,Wilks' Lambda = 0.89], and a significant interaction between educational level and test time [*F*_(2, 231)_ = 3.42, *p* = 0.034,Wilks' Lambda = 0.71]. As it can be seen in Figure 8, the 4th EP gave higher ratings, and there were also higher ratings in post-test than in pre-post.

In order to explore the significant interaction between educational level and test time, separate MANOVAs for each educational level were conducted with within-subject factor test time and with between-subject factors sound emotion and sound spatiality conditions. The results of the multivariate test revealed that there was a significant main effect of test time for the 4th EP [*F*_(2, 115)_ = 12.53, *p* < 0.001,Wilks' Lambda = 0.82] and for the 1st ESO [*F*_(2, 115)_ = 4.84, *p* = 0.010,Wilks' Lambda = 0.92]. Univariate tests revealed a significant pre-post effect on valence for both educational level, the 4th EP [*F*_(1, 116)_ = 15.22, *p* < 0.001], and the 1st ESO [*F*_(1, 116)_ = 9.76, *p* = 0.002] as well as a significant pre-post effect on arousal only for the 4th EP [*F*_(1, 116)_ = 23.27, *p* < 0.001]. The rest of univariate tests were not significant. Overall, participants from both educational levels reported more pleasant emotional state in the post-test than in the pre-test, and participants in the 4th EP group reported being more aroused in the post-test than in the pre-test. For all analyses, there was no significant effect of the sound emotion condition or sound spatiality condition, and neither was there an interaction between the sound condition and test time (all ps > 0.05).

### Self-Report of Immersion Level

**Figure 9:**
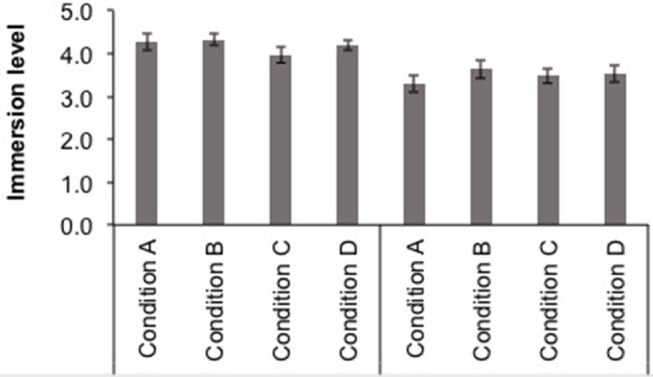
Mean immersion ratings ± SE (on a five-point scale) for each course (4th EP, on the left, and 1st ESO, on the right) and sound condition (A-Neutral stereo, B-Neutral 3D, C-Emotional stereo, and D-Emotional 3D).

The hypothesis proposes that the immersion level will be higher in the sound conditions where a 3D sound mix has been used. A specific question was presented to the participants—“Have you felt that you were inside the story?”—using a Likert scale with five answer options (None/A little bit/Some/Quite/A lot). The mean immersion ratings for each course and sound condition (A—Neutral stereo, B—Neutral 3D, C—Emotional stereo, and D—Emotional 3D) are displayed in Figure 9.

First, the immersion ratings were entered into a Kruskal-Wallis test, one for each educational level, to investigate potential modifications between the four sound conditions. This analysis showed no significant differences between conditions (*p* > 0.05). Then, in order to test the potential interaction between the factors sound emotion and sound spatiality, the immersion ratings were used as dependent variables for two ANOVAs, one for each educational level, with between-subject factors sound emotion condition (Neutral and Emotional) and sound spatiality condition (stereo, 3D). These analyses did not yield significant results (*p* > 0.05).

A positive correlation between SAM test and immersion level has been found, using Spearman' rho statistics: higher valence and arousal correspond to higher immersion level (see Table 3).

**Table 3:** Correlations (Spearman' rho values) between Immersion, Valence, and Arousal for the two pre-test and post-test measures.

**Variables**	**1**	**2**	**3**	**4**	**5**	**6**	**7**
Valence—Pretest 1	1						
Arousal—Pretest 1	0.522^**^	1					
Valence—Pretest 2	0.529^**^	0.444^**^	1				
Arousal—Pretest 2	0.389^**^	0.683^**^	0.551^**^	1			
Valence—Post	0.520^**^	0.402^**^	0.619^**^	0.401^**^	1		
Arousal—Post	0.411^**^	0.578^**^	0.453^**^	0.620^**^	0.648^**^	1	
Immersion	0.307^**^	0.227^**^	0.313^**^	0.288^**^	0.449^**^	0.413^**^	1

** *Correlation is significant at the 0.01 level (2-tailed)*.

### Effects on Perceived Emotions

The hypotheses propose that the different sound conditions will generate distinctive intensity levels of perceived emotions in the listener. Two specific questions were asked to the participants after listening to the story:

- What moment in the story did you find most exciting? Why? (Q1)
- How did you feel at that moment? (Q2)

The answers to both questions were registered using the Think-aloud technique. The qualitative analysis of the recordings showed that the different sound conditions generated distinctive reported emotional responses in the participants. Several categories were established: moment of history that seemed most exciting; the type of emotion that this moment generated in participants; and the intensity of that emotional response.

**Figure 10:**
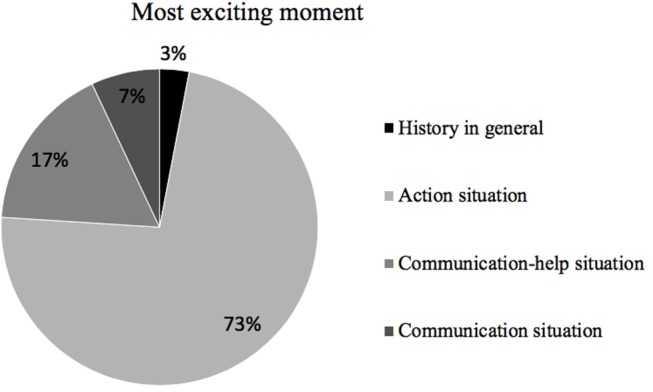
Distribution of the most exciting moment between the identified subcategories.

In relation to the moment of history that they found most exciting, four subcategories have been identified for this dimension of analysis: history in general; action situation; communication–help situation; and communication situation. The one that stood out with a high percentage of references was the one related to an action situation, followed by the communication–help situations, as shown in Figure 10.

The moments of action narrated mainly focus on the episode in which Elena throws herself on the ramp with the skateboard, showing in most cases the emotion through expressions of intensity, onomatopoeia, or narrating unexpected and catastrophic outcomes.

“*Elena throws herself with the skateboard from the slope of the school and began to do with her mouth buaaah*.” (Condition A\\263_PPBB-4EP-B-05)

“*Well, I felt that she was going to throw herself for sure—that she was going to crash, and that in the end she had time to play a fragment of the flute—because…* (it is not understood) *if she enters by the flute and if she plays the flute is impossible to play, it can play*.” (Condition A\\302_PPBB-4EP-D-05)

“*Elena fell with the skateboard because it was a very steep slope and she had to be very careful and I thought she was going to be in a coma and she was going to breathe running and that…*” (Condition A\\625_SSAA-1ESO-A-03)

“*When she threw himself down the steep and so big slope and when she put on the cow's face, because that part was so funny, I loved it and it was very cool, I want to do it, I love it, I love it. Well the truth is that I did not like it, well I liked everything…” (*Condition C \\ 332_PPBB-4EP-A-14)

**Figure 11:**
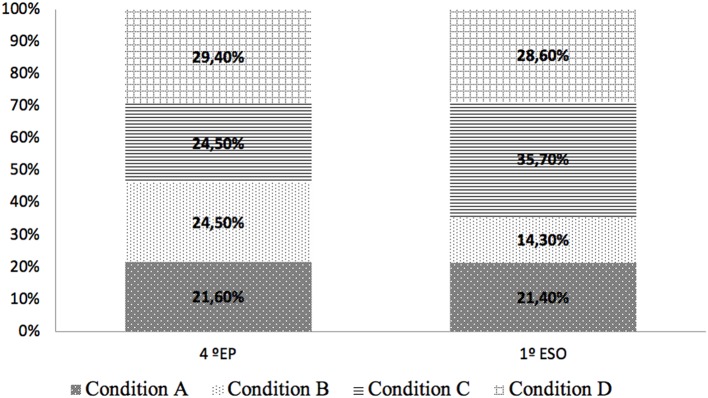
Perceived emotion in action situations comparison between 4th EP and 1st ESO participants.

Focusing on those situations of action of the story that has elicited more intense emotion in the participants, no significant differences have been found between the two educational levels (4th EP or 1st ESO). As shown in Figure 11, in both educational levels, the conditions that collect more fragments in this subcategory are those that correspond to an arousal sound treatment: condition C and condition D. In the 1st ESO participants, condition C was the one that generated a greater amount of emotions in action situations, while in 4th EP participants it was the condition D condition. In those passages in history that relate moments of action, e.g., when Elena throws herself on the ramp with the skateboard or when Salva disguises himself, the arousal treatment of the sound contributes to enrich the description and enhance the action.

In relation to the type of emotions that participants felt while listening to the story, four subcategories of analysis have been identified and defined:

- Positive: expressions that produce participant well-being.
- Negative: expressions that generate participant discomfort.
- Neutral: expressions that do not show reactions either pleasant or unpleasant.
- Contradictory: those expressions where the manifestation of the same feeling by the participant reflects conflicting emotions (positive or negative).

**Figure 12:**
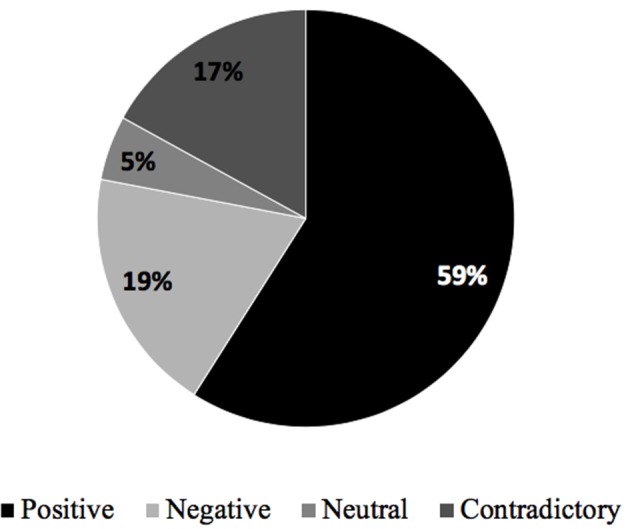
Type of emotions felt by participants.

Participants state a high percentage of expressions of emotions, feelings, or positive moods throughout their responses, as shown in Figure 12. Negative and contradictory emotions also have a significant presence in this category of analysis.

**Figure 13:**
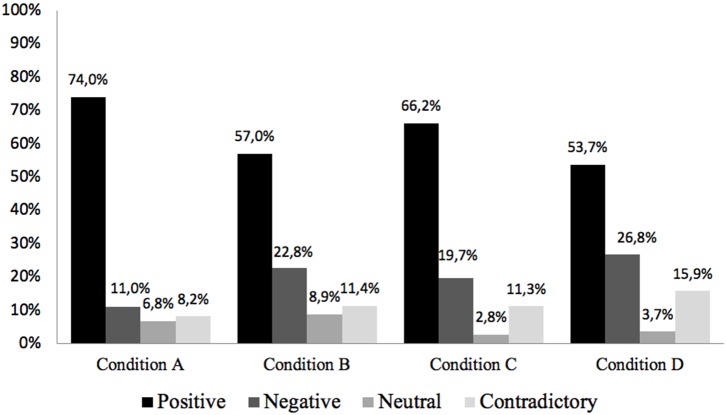
Type of emotion per sound condition (%).

Depending on the type of condition to which the participants have been exposed while listening to the story, the “Positive” emotions exhibited a higher presence in all four types of conditions, as Figure 13 shows. The results in this category do not show a significant difference between participant groups either. The condition A stands out, especially with 74%, with a minimum presence of the rest emotions. However, the condition D is the condition that generates in the participants a greater variety of emotions, highlighting the positive, negative, and contradictory types of emotions. According to this result, it is concluded that the condition that combines the two sound treatments (arousal sound design + 3D sound mix) is the one that generates a greater diversity of emotional responses (positive, negative, contradictory, or neutral) in the listener.

The emotional responses felt by the participants, apart from being classified according to a type of emotion, can have greater or lesser intensity (Supplementary Figure 1). Most of the emotions raised by the listening of the story in the participants are classified as “High intensity.” No significant differences have been found between the four conditions, just minimal variations between them.

This “High intensity of emotions” is expressed by participants through adverbs of quantity, profusion in the manifestation of various emotions, or biological responses to stimuli (e.g., laughter):

“*I felt super cool.”* (Condition A\\263_PPBB-4EP-B-05)

“*I have felt happy, imaginative. I have felt excited, passionate, deep within the story ehh thoughtful.”* (Condition A\\277_PPBB-4EP-B-03)

“*I had a great time.” “It was very cool.” “It made me very funny.” “It made me laugh a lot.” “Very funny, and I laughed a lot because, because I liked it a lot.”* (Condition A\\297_PPBB- 4EP-D-02)

“*I felt very laugh. I felt super, super lively as if it were a humorous story, super cool story, very exciting story.”* (Condition B\\269_PPBB-4EP-B-06)

“*I laughed a lot with them because I liked it a lot, and I loved this story because it is so much fun.”* (Condition B\\339_PPBB-4EP-A-12)

“*I felt happy, funny, entertaining, imaginative.”* (Condition C\\320_PPBB-4EP-C-13)

“*I felt very happy.”* (Condition D\\633_SSAA-1ESO-A-19)

“*I have felt super excited, and I really liked the story.”* (Condition D\\672_SSAA-4EP-A-02)

### Mental Images Elicited by the Story

The proposed hypothesis (hypothesis H4) that both quantity and richness/detail of images will be higher in the CD sound condition than in the other conditions. According to this hypothesis, two dimensions were measured. On one side, the number of mental images elicited by the story and, on the other hand, the richness and detail of such images.

The collected data from the think-aloud technique, as answers to two of the proposed questions to participants, were used to measure both dimensions:

- Try not to think too much, and speak as things come to your mind: what have you imagined when Elena was going down the school hill on the scooter? (Q3)
- Describe how you have imagined Salva (Q5)

The qualitative analysis of the information collected from Think-aloud was carried out according to two main categories: description of the represented situations and the number of elicited images when they narrate an episode of the story.

In the analysis carried out on the description made by the participants of the situations that tell about the story three subcategories have been inferred depending on the content they narrated: as it is told in the story; invented but same plot and characters; or invented with a changed argument but with elements present in other passages. According to the condition of the stimulus to which the participants have been exposed, condition D is the one that has generated the recreation of mental images more faithful to what is told in the story (Supplementary Figure 2).

This finding confirms the hypothesis that an “emotionally marked” sound design in the context of a sound story will generate richer and more detailed mental images in the listener than a soundtrack without this emotional purpose.

Per educational level, 4th EP participants exposed to Conditions with arousal treatment (C and D) have elaborated richer and more detailed mental images compared to the ones who listened the neutral conditions. On the other hand, in the case of 1st ESO participants, it was the Condition B the one that generated more detailed and richer mental images.

In general, the mental images that the participants represent are present in the story—specifically in two key moments of the story, the scene of Elena going down the street over a skateboard and the scene in which Salva dresses himself as a scientist—as are passages invented by the participants themselves.

“*The part where she was thrown by, was pulling crazy on the ramp has been very cool, but the image of Salva's dressed as a scientist has come to mind. It has also come to mind and that I found it very funny because he looked like a madman, a mad scientist.”* (Condition A\\274_PPBB-4EP-B-04).

“*Elena—well, Salva's sister whose name was Elena—doesn't know how to play the flute, and Salva the brother went to comfort her, invents words to make her laugh, told her to play a piece of …(can't be heard) with the flute.”* (Condition B\\654_SSAA-1ESO-B).

“*Well, at first there were the two brothers in the town. They went uphill, and I was imagining, I was imagining that it was a town, and I was imagining the car races, and also the stops in a pile of a hill, and I also imagined the moment when the sister was sad, and then it seemed funny when the brother took the diapers, the little children diapers, and now takes a costume, well the costume from the grandmother, and then he puts it on, and then he does experiments, does experiments until he success, come on, that the brother is very crooked. The truth is that he is very cool. I would love to hear it again*.” (Condition D\\323_PPBB-4EP-C-21).

Finally, the number of mental images that the story provokes in the participant has been measured depending on the sound condition. As Supplementary Figure 3 shows, Condition B and Condition C are the conditions that generate the greatest number of mental images in the participants when they tell and recreate the story. As results show, in the 4th EP educational level, the arousal condition shows a higher amount of mental images but only in the stereo condition. Otherwise, in the 1st ESO educational level, the 3D sound condition shows higher number of mental images than stereo condition, though only in the neutral condition.

Finally, to complement the qualitative analysis, two further closed questions were included to quantitatively measure the kind and number of mental images:

- “The images that have come to your mind while you were listening were:”
  ∘ Real—Fictitious (four-point Likert scale)
  ∘ Clear—Unclear (four-point Likert scale)
  ∘ Exciting—Boring (four-point Likert scale)
  ∘ Known—Unknown (Q6) (four-point Likert scale)
- “After listening to the story … images came to my mind.” Multiple option question:
  ∘ A lot of
  ∘ Some
  ∘ A few (Q7)

Responses to each of these questions were used as dependent variables for an ANOVA with between-subject factors educational level (4th EP and 1st ESO), sound emotion condition (Neutral and Emotional), and sound spatiality condition (stereo and 3D). Results showed only a main effect for the questions related to clear/unclear images [*F*_(1, 231)_ = 5.08, *p* = 0.025] and exciting/boring images [*F*_(1, 231)_ = 33.28, *p* < 0.001]. Non-parametric Kruskal-Wallis were run to confirm the significant effect in relation to clear/unclear images [H_(1)_ = 5.72, *p* = 0.017] and exciting/boring images [*H*_(1)_ = 31.97, *p* < 0.001]. In a scale ranging from 1 (clear) to 4 (unclear), 4th EP students found the images more unclear (M = 3.32, SE = 0.08) than 1st ESO students (M = 3.05, SE = 0.08). Furthermore, in a scale ranging from 1 (exciting) to 4 (boring), 4th EP students also found the images more boring (M = 3.75, SE = 0.06) than 1st ESO students (M = 3.25, SE = 0.06).

## Discussion

As exposed in the results section [Effects on Physiological Arousal (EDR)], the means of the physiological analyses do not show significant differences between conditions.

When considering the moments that have a specific sound treatment, a non-significant tendency indicated an interaction between sound emotion condition and sound spatiality condition. Overall, there was higher EDR for the “emotional” than for the “neutral” conditions, and only for the “neutral” conditions was the expected increase in EDR from the “stereo” to “3D” condition observed. When looking separately at the two educational levels, 4th EP and 1st ESO, we found differences between them. In all these moments, a constant pattern is found in the 4th EP participants: the arousal condition shows a higher impact of the EDR, confirming the initial hypothesis (H1), but only in the stereo condition. In 3D conditions, the variance in the impact of the arousal condition over the neutral is not so remarkable. Otherwise, in the 1st ESO participants, the 3D sound condition obtains higher EDR levels than stereo condition, but the evolution between neutral and arousal is not so consistent.

On the other hand, upon analyzing hypothesis H3—“a soundtrack mixed in 3D sound format will elicit more intense emotional response in the listener compared to a soundtrack mixed in stereo”—it was found that, as results show, 1st ESO participants are more affected by the 3D sound than by the arousal treatment of sound even in the moments in which there is no special 3D sound treatment. In the moments where the three-dimensionality of the sound is more focused, the higher impact of the 3D sound condition is more evident in both educational levels, which reinforces previous findings (Murphy and Pitt, 2001; Steele and Chon, 2007) and reinforces the initial hypothesis (H3). However, in 4th EP participants, the EDR level of the 3D condition falls when combined with arousal treatment. We hypothesized that this disparity between educational levels may be explained by the fact that it is a “sound only” stimulus, and a greater level of cognitive maturity may be necessary to decodify both processes (arousing and spatialization), which indicates that the impact in younger children is greater in the arousal condition where the sound treatment becomes more evident than the 3D sound mix. This hypothesis needs further investigation in future research, as there are no previous studies on sound emotional impact on different ages that can strength or refute this argument. Furthermore, while older people may be more habituated to the arousal treatment of sound, as it is a very common process used in film sound production, 3D sound is quite a novel narrative technique that may have a greater impact, which may clarify and justify the greater level of emotional impact of 3D condition over stereo and over neutral or arousal treatment.

Results from the Think-aloud analysis (subsection Effects on Perceived Emotions) confirm most of the findings obtained from EDR and SAM measurements. On the one hand, those moments in the story in which both arousal sound treatment and 3D sound mix have been applied are the ones that students stand out as the most exciting ones: action situations (Elena on the skate down the street) and communication–help situations (Salva dressing as a scientist). Although no significant differences have been found between conditions, in the case of action situations, the arousal predominant condition (condition B) has had a greater emotional impact on the 4th EP participants, while in the 1st ESO students, the 3D sound condition is the one that obtains the greatest emotional impact when identifying action situations throughout the story. These results are consistent with the EDR response patterns in both educational levels: younger participants are more affected by the arousal treatment, while 3D mix has a greater impact over older participants.

On the other hand, regarding the valence and intensity of the reported emotions, a high percentage of positive emotions, feelings, and moods have been identified in the responses without finding significant differences between the participants of both educational levels or between the four conditions. In accordance with these results, there is consistency between the data obtained in the quantitative and qualitative analysis, confirming hypotheses H1 and H3.

Another of the elements proposed for analysis was related to H2—“the use of emotional marked sound in the design and production of a sound story will generate richer and more detailed mental images in the listener than a soundtrack without this emotional intention”—and H4—“a soundtrack mixed in 3D sound format will generate richer and more detailed mental images in the listener compared to a soundtrack mixed in stereo.”

In relation to the mental images reported through the Think-aloud results, in those cases in which the students have been exposed to 3D sound mix conditions, the description they made regarding the scene of Elena's descent down the street on the skateboard was that it been more real and detailed in accordance with the story. This finding confirms the H4 hypothesis. It is also noted that sound condition B has generated a greater impact on the number of reported mental images in 4th EP students. However, for the 1st ESO students, it is the treatment of 3D sound that caused a greater number of mental images in students. The pattern identified in the EDR response between the different groups is also maintained in the analysis of the mental images.

Finally, according to H5—“the emotional impact elicited by both emotional marked sounds and/or 3D sound mix format takes place at an unconscious level and is not self-perceived by the listener”—and regarding self-reported emotions, we found that, overall, participants from both educational levels reported more pleasant emotional state in the post-test than in the pre-test, showing that they liked the experience. Furthermore, participants from 4th EP reported being more aroused in the post-test than in the pre-test, showing that they also found the story exciting. The fact that the 1st ESO students did not show this change may be explained by the story being more suitable to the younger students. There were no differences due to the sound treatment in self-reported emotions or immersion level, that is, in the answer of the participants related to their own perception of the emotion level and the immersion level. Otherwise, there were differences in the other measures of the emotion and immersion levels (not self-perception but physiological, amount, richness, and detail of mental images). These results confirm hypothesis H5: the emotional impact elicited by both emotional marked sounds and/or 3D sound mix format takes place at an unconscious level and will therefore not be reported by the listener.

## Conclusions

As a main conclusion, the hypotheses have been partially confirmed. Although no significant correlations have been found between the conditions and the variables considered, different patterns depending of educational levels (and, subsequently, age of participants) have been identified. This opens the topic to further studies in which age (and diverse aspects related to this parameter, much like cognitive development or consumer habits) must be considered in the definition of variables.

Apart from the results obtained analyzing the different variables independently, a relevant finding has emerged from the combination of the two variables (arousal sound treatment and 3D audio mix). The interaction between these variables in the four conditions generates a different response in the participants, particularly in EDR measurement, than the response obtained when only one of the variables is considered.

Some limitations must be considered from the present study:

- First of all, the subtlety and diversity of the sound treatment: with the purpose of giving a step forward in the research field, the differences between conditions have been based on specific changes in the sound that are quite subtle and not consciously perceived by the listener rather than being based on the intervention on more noticeable changes, such as the presence or absence of certain elements. Otherwise, this approach has also made possible to carry out a field study with listening conditions as close to real situations as possible.
- In relation to the aforementioned, the combination of variables in the same stimulus (emotional-marked or neutral with stereo or 3D sound) may have limited the clarity of the results because of the interaction of these variables, as has been detailed in relation to the different cognitive development of the diverse educational level participants, but also considering other contextual factors that refer to the social and cultural memory, such as previous experiences or expectations, which is consistent with previous findings (Tajadura-Jiménez, 2008; Grimshaw, 2014).

Finally, further developments and applications of the present study are proposed:

- Replication of the present study with wider age range and extending the sample to adults.
- Studies derived from the present one, but focused on specific sound treatment, in order to enrich and complete the knowledge base about the emotional impact of different sound with real-world stimulus and listening situations.
- Application of the results to the production of sound stories, but also to video games, films, or advertising. As Dafonte-Gomez (2014, p. 206) concludes in another study on viral advertising, “the obtained results show the outstanding presence of surprise and joy as dominant emotions in the most successful viral videos.” According to the results of the present study, this kind of positive response can be achieved through the use of arousal sound treatment.
- Application in the educational environment: as Mora (2005) states, an emotionally marked experience is best remembered, especially because of the connection between the hippocampus and the amygdala, where our emotions are represented. A sound-based educational resource focused in the arousal treatment of the sound may improve the learning experience. With this purpose, a first development from the present study has been carried out: as part of the “Unconscious listening” project “Gale's journey,” an educational project based on the use of arousal sound treatment and TUI object interface, has been designed to foster the teaching of different contents from the Primary Education curriculum in Spain: Natural Sciences, Social Sciences, EFL, and Music. A first exploratory study has also been carried out, and its results are reporting a high level of usability (easy to use, clear, and appealing) as well as positive student feedback in terms of motivation, attention level, and learning improvement.

## Data Availability Statement

The datasets generated for this study are available on request to the corresponding author.

## Ethics Statement

The studies involving human participants were reviewed and approved by Comité de Ética de la Universidad Loyola Andalucía. Written informed consent to participate in this study was provided by the participants' legal guardian/next of kin.

## Author Contributions

FC and AT-J described the theoretical framework and were in charge of the literature search. FC wrote the Methods section. IL-C and AT-J were in charge of the quantitative analysis and results, while TM-B was in charge of the qualitative analysis and results. All authors participated in the discussion and elaborated the Hypothesis section.

## Conflict of Interest

The authors declare that the research was conducted in the absence of any commercial or financial relationships that could be construed as a potential conflict of interest.

## Supplementary Material

Supplementary Figure 1 Intensity of perceived emotions.

Supplementary Figure 2 Subcategories of mental images elicited by the four sound conditions.

Supplementary Figure 3 Number of mental images elicited by the four sound conditions.

Supplementary Audio 1 Meteor_fall_neutral.mp3.

Supplementary Audio 2 Meteor_fall_arousal.mp3.

Supplementary Audio 3 Chronometer_neutral.mp3.

Supplementary Audio 4 Chronometer_arousal.mp3.

Supplementary Audio 5 Scientist_st.mp3.

Supplementary Audio 6 Scientist_3D.mp3.

Supplementary Audio 7 Skate-fall_st.mp3.

Supplementary Audio 8 Skatefall_3D.mp3.

## Appendix 1. Apparatus

The physiological measurement of electrodermal activity was made using the Sociograph measuring instrument, patent n° 9902767. It has been used in previous studies, such as those of Martínez Herrador et al. (2008, 2012), Aiger et al. (2013), Tapia and Martín (2015, 2016).

For sound story playblack and data collection (answer to questionnaires and think-aloud recording), an individual 8 Android tablet was used for each participant. It had the following technical specifications

- Manufacturer and model: BQ, Aquaris B8.
- Screen: 8, Resolution: 800 × 1200, 189 ppi, Aspect ratio: 16:10
- Processor:
  ∘ CPU: MediaTek Quad Core MT8163B, 1,3 GHz
  ∘ GPU: MediaTek Mali-T720 MP2, 520 MHz
- RAM: 2 GB.

The headphones used were overhead models with microphone, and they had the following specifications

- Manufacturer and model: Mars Gaming, MH2
- Speaker diameter: 40 mm
- Speaker Impedance: 32 Ohm
- Frequency response: 20 Hz–20 KHz
- Maximum input power: 20 mW
- Sensitivity (SPL): 105 dB +/– 3 dB
- Microphone Dimensions: Dia 6.0 × 5.0 mm
- Sensitivity: –54 db +/– 3 dB
- Directivity: omnidirectional
- Impedance: <2.2 KOhm
