# Supplementary figures and images for: Arousing the Sound: A Field Study on the Emotional Impact on Children of Arousing Sound Design and 3D Audio Spatialization in an Audio Story
Source: Front Psychol. 2020 May 6;11:737. doi: 10.3389/fpsyg.2020.00737 (PMC7219267; doi:10.3389/fpsyg.2020.00737)

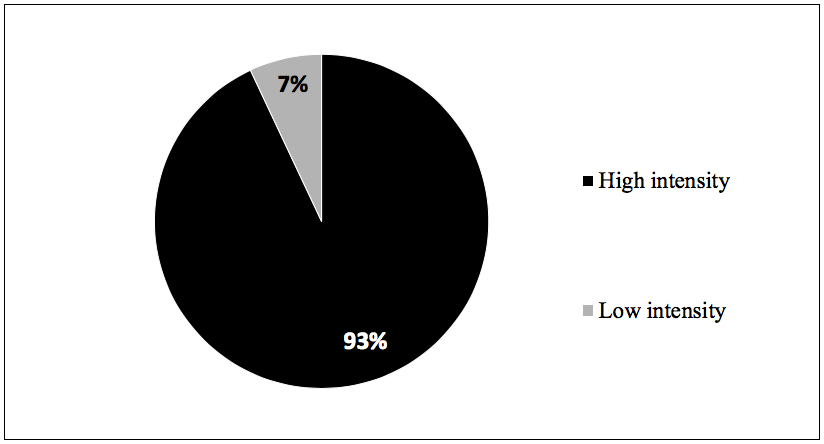

Supplement: Supplementary Figure 1 — Intensity of perceived emotions. [file Image_1.JPEG]

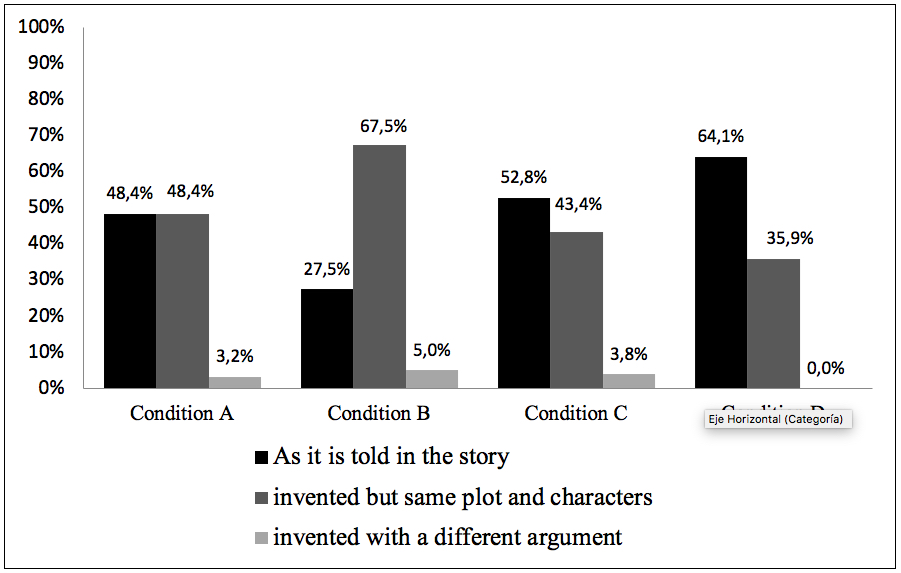

Supplement: Supplementary Figure 2 — Subcategories of mental images elicited by the four sound conditions. [file Image_2.JPEG]

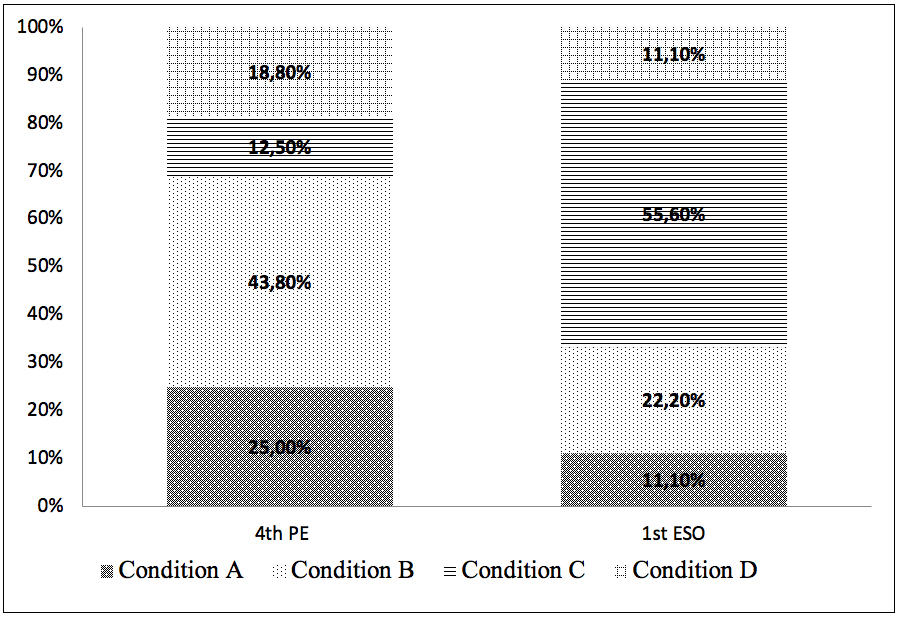

Supplement: Supplementary Figure 3 — Number of mental images elicited by the four sound conditions. [file Image_3.JPEG]
